# The Strike at Shane’s

**Published:** 1894-03

**Authors:** 


THE STRIKE AT SHANE'S. Gold Mine Series, No. 2. A sequel to "Black Beauty," a prize story of Indiana, written for, and revised, copyrighted, and published by the "American Humane Education Society." George T. Angell, President, 19 Milk Street, Boston, Mass. Price, 10 cents.

This Society has published and caused to be circulated nearly a million and a half copies of "Black Beauty." "This little story is intended to point out in a homely way some of the mistaken ideas held by men in general in regard to the relation existing between the human race and the lower animals and birds." The book is intended to show the results that would naturally follow if the support and assistance given us by the lower animals and birds should be withdrawn, as would be the case if they should exercise the same rights claimed by human toilers and go on a strike. This book can be obtained by writing to George T. Angell, President, 19 Milk Street, Boston, and inclosing 10 cents in stamps.

